# Using Twitter to Better Understand the Spatiotemporal Patterns of Public Sentiment: A Case Study in Massachusetts, USA

**DOI:** 10.3390/ijerph15020250

**Published:** 2018-02-02

**Authors:** Xiaodong Cao, Piers MacNaughton, Zhengyi Deng, Jie Yin, Xi Zhang, Joseph G. Allen

**Affiliations:** 1 Department of Environmental Health, Harvard T.H. Chan School of Public Health, 401 Park Drive, Boston, MA 02215, USA; xcao@hsph.harvard.edu (X.C.); piers.macnaughton@gmail.com (P.M.); zhd471@mail.harvard.edu (Z.D.); jieyin@g.harvard.edu (J.Y.); annezhang1129@sjtu.edu.cn (X.Z.); 2 School of Naval Architecture, Ocean and Civil Engineering, Shanghai Jiao Tong University, Shanghai 200240, China

**Keywords:** Twitter, sentiment analysis, land use, social media, happiness

## Abstract

Twitter provides a rich database of spatiotemporal information about users who broadcast their real-time opinions, sentiment, and activities. In this paper, we sought to investigate the holistic influence of land use and time period on public sentiment. A total of 880,937 tweets posted by 26,060 active users were collected across Massachusetts (MA), USA, through 31 November 2012 to 3 June 2013. The IBM Watson Alchemy API (application program interface) was employed to quantify the sentiment scores conveyed by tweets on a large scale. Then we statistically analyzed the sentiment scores across different spaces and times. A multivariate linear mixed-effects model was used to quantify the fixed effects of land use and the time period on the variations in sentiment scores, considering the clustering effect of users. The results exposed clear spatiotemporal patterns of users’ sentiment. Higher sentiment scores were mainly observed in the commercial and public areas, during the noon/evening and on weekends. Our findings suggest that social media outputs can be used to better understand the spatial and temporal patterns of public happiness and well-being in cities and regions.

## 1. Introduction

Social media has become ubiquitous in daily communications. Twitter is currently the most popular social media platform, with a global reach of 1 billion monthly visits to the site with embedded tweets by 313 million active users [1]. Roughly 82% of active users access the service via mobile devices [1], giving a good opportunity to track the geographical location and time of the posted tweets. Consequently, Twitter can provide a large amount of spatiotemporal information about the users broadcasting their real-time opinions, sentiment, and activities.

Geo-tagged tweets have been widely used in geographical information system (GIS) research. GIS researchers are particularly interested in studying the location awareness and social characteristics based on collected tweets [2,3,4]. Though providing new insights into using georeferenced tweets, these studies were more focused on the locational property rather than the textual components of tweets. Further, tweets can record users’ daily activities varying across personal characteristics, locations, and temporal rhythms. Such variations are unlikely to be discovered by conventional geodemographic methods, which associate activities only with residence at nighttime [5]. Along with topic modeling techniques [6], the geographic distribution of Twitter data can further help to understand the social dynamics in urban areas [7]. For example, Lansley and Longley [8] systematically studied the geography of Twitter topics in London, and found topics expressed through tweets varied significantly across different land uses and times in Inner London. The mobility patterns of Twitter users can also be used to classify urban land use types with reasonable accuracy [9]. These studies demonstrated the feasibility of tracking social trends across time and space with Twitter data.

In addition to spatiotemporal information, the contents of tweets also provide substantial information regarding users’ opinions and sentiment. Quantifiably interpreting tweets relies on employing sentiment analysis [10], which is used to computationally translate opinions and expressions of human sentiment into data that can be quantified and categorized [10]. Specifically, a major focus of sentiment analysis tools is identifying the positive, neutral, or negative polarity of a given text [11]. Identification of the polarity by Twitter users has been used to study the geographical property of public sentiment during Hurricane Sandy [12]. Extracting sentiments during disaster events can help develop stronger situational awareness of the disaster zone. Jiang et al. [13] used sentiment analysis to systematically assess the online public onions on an ex-post evaluation of a large infrastructure project in China. Yu and Wang [14] performed sentiment analysis to examine U.S. soccer fan’s emotional reactions towards the World Cup 2014 games from their tweets. They found strong relationships between the fan’s sentiment, participated teams, and goal results. Palomino et al. [15] conducted sentiment analysis for 175,000 tweets related to the natural-deficit disorder. They concluded that the dissemination of nature-health concepts was associated with both the hashtags used and the sentiment of the message. A recent study applying sentiment analysis of geo-tagged tweets about food revealed the prevalence of healthy and unhealthy food across the continental USA [16]. Their findings can be used to identify regions that have low access to healthy food.

As exemplified in these research examples, Twitter data are increasingly perceived as “social sensors” to better understand the social phenomenon in the real world [17]. Twitter data have already been used to provide valuable information about public issues on myriad topics, e.g., outdoor air pollution [18], opinion polling [19], stock markets [20], and elections [21]. There is also a growing interest in Twitter-based approaches for public health research [22]. One important use of Twitter data in health-related research is disease surveillance and prevention, such as influenza outbreaks [23] and HIV prevalence [24]. These studies indicated that the epidemic spreading in the real world can be well traced in relevant tweets. A recent review paper [25] proved that sentiment analysis of Twitter data has been used in many different domains to solve various problems. The greatest value of this discipline is to extract the intrinsic knowledge disseminated in tweets to help government or business intelligence.

In addition, there has been an increasing number of studies that use Twitter data to measure and analyze the quality of life (QoL) and happiness in cities and regions, aiming to find out what makes a “happy city” [26]. QoL is a measure of social well-being and life satisfaction of people in cities/regions. Most of the efforts are still based on objective measures of QoL, including income, housing, urban land use, natural environment, environmental pollution, and local amenities, etc. These objective indicators are relatively easy to be quantified for ranking the urban and regional QoL [27,28]. On the other hand, increasing attention has also been paid to the evaluation of subjective QoL, such as subjective happiness and well-being. Previous quantitative studies of happiness are normally based on subjective measures of well-being derived by questionnaire surveys [29,30]. Oswald and Wu [31] explored the life satisfaction in each state based on the results from U.S. Behavioral Risk Factor Surveillance System. They confirmed a state-by-state match between subjective and objective well-being. Recent studies already suggest that while most of the variations in subjective well-being are attributable to individual characteristics, some of the variations can also be associated with the geographical context and regional factors [32,33]. It is necessary to investigate the impact of geographical context upon subjective QoL. Bhatti et al. [34] spatially examined the relationships between surveyed QoL, land use and population density in an urban environment of Lahore. An inverse relationship was observed between QoL with built-up and population densities. Some researchers also studied the geographical distributions of subjective QoL in cities based on GIS approaches [35,36,37]. These studies proved that reasonable planning of land use was an important factor for building a successful city with both high objective and subjective QoL. 

As introduced above, Twitter data provide a more economical and dynamic approach compared to traditional population survey and, more importantly, an ability to add objective geometrical dimension to the subjective study of QoL. It is being recognized that there is a great potential in identifying key spatial characteristics and factors of cities and regions pertaining to subjective well-being measures. The users’ sentiment expressed by tweets is likely to vary in different surrounding environments and time periods, according to the user’s activities and opinions. Some studies have been carried out combining sentiment analysis, spatiotemporal analysis, and domain knowledge for public well-being. Yang and Mu [38] applied GIS methods to Twitter data to detect clusters of major depressive disorder users. They provided an alternative way to diagnose depression in a large population. Nguyen et al. [39] built up a neighborhood dataset of happiness, diet, and physical activity across the 2010 census tracts of the U.S. based on a large corpus of tweets. They concluded that the tracts with the social and economic disadvantage, high urbanization, and more fast food restaurants may exhibit lower happiness and fewer healthy behaviors. Mitchell et al. [40] also studied the geography of happiness across all 50 U.S. states based on a large dataset of tweets. Happiness within each city/state was found to be positively correlated with wealth and anti-correlated with obesity rates. Another group of researchers investigated the weekly trend of emotion and work stress by Twitter analysis [41]. The linguistic inquiry word counts indicated a clear “Friday dip” for work stress and negative emotion tweets and a “weekend peak” for positive emotion tweets. 

Current studies mainly focused on the descriptive analyses of users’ polarity and mobility patterns. However, less attention has been paid to the spatiotemporal patterns of users’ sentiment scores by using statistical analyses. To address this issue, we emphasized both the land use/time aspects and the quantified sentiment scores from Twitter data in this study. In this paper, we used Twitter data along with high-resolution land use data to reveal the spatiotemporal variations of public sentiment, through a corpus of nearly one million of georeferenced tweets collected within Massachusetts (MA), USA. The MassGIS data were used to classify the land use categories. Computational sentiment analysis was employed to quantitatively identify the users’ polarity with sentiment scores from the collected tweets on a large scale. We further used a multivariate linear mixed-effects model to statistically reveal the prevalence of users’ sentiment across different geographical locations and time periods. This case study demonstrated an economical approach to investigate the spatiotemporal patterns of subjective QoL of Twitter users in cities and regions. 

## 2. Materials and Methods

### 2.1. Twitter Data 

The study is based on a Twitter dataset of 880,937 tweets posted by 26,060 users within Massachusetts, USA from 31 November 2012 to 3 June 2013, collected via the Twitter streaming API (application program interface). Tweets were collected within a one-mile radius of all private and public schools in Massachusetts (kindergarten to grade 12, n = 2613 [42]). The one-mile radius of these schools can basically encompass all urban/suburban areas in Massachusetts. All the collected tweets were restricted to those with precise geographical locations, post time, original content in English, and active users posting more than 10 and less than 270 tweets (equivalently up to 1–2 tweets/day) within the timeframe. This restriction aimed to avoid non-active users with fewer than ten posts and hyperactive users that often belong to commercial entities or twitter bots. We restricted the criteria to geo-tagged tweets to gain the geolocation of the sampled tweets as accurate as the mobile phone’s location, which is accurate to about a range of 8 m [43].

**Figure 1:**
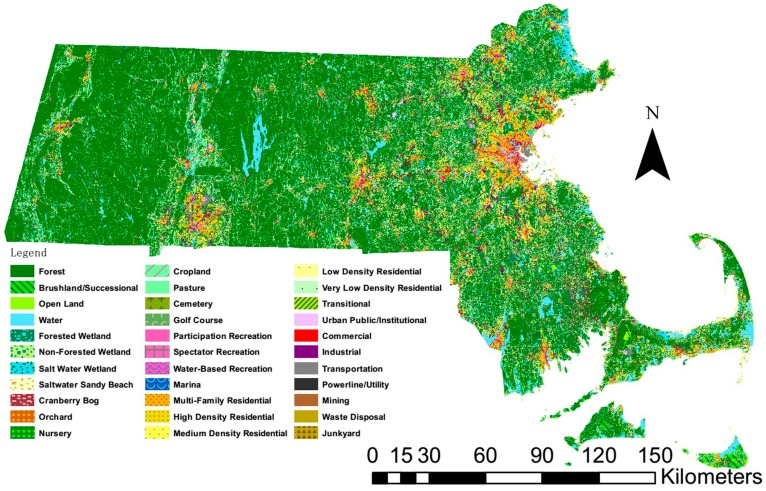
Geographical distribution of 33 land use types within MA (mapping based on the MassGIS land use data [44]).

The geotagged tweets were mapped out with the land use data by using the ArcGIS Desktop 10.4.1 (Esri, Redlands, CA, USA). Then the land use types were correspondingly attributed to those tweets which fell within their polygons based on the geographical coordinates. Figure 1 shows the geographical distribution of 33 land use types in MA, mapping based on the MassGIS land use data [44]. The data layer contains a Massachusetts statewide, seamless digital dataset of land cover/land use, created using semi-automated methods, and based on the 0.5-m resolution digital orthoimagery captured in April 2005. The minimum mapping unit (MMU) is generally one acre, but an MMU as low as 0.25 acre may be found in some urban areas. A more detailed definition of each land use type can be found on the MassGIS website [44]. The land use types were further grouped into eight categories according to their intra-similarities, as shown in Table 1. The cemetery, nursery and transitional land uses were excluded from analysis due to their limited sample sizes. The land of MA was largely covered by the land use category of nature, followed by the residential and commercial land uses that are typically concentrated in the urban/suburban areas. The collected number of tweets (Table 1) was correspondingly related to the coverage of each land use category.

The temporal information of the tweets was also classified for the statistical analysis. The time periods of a day were classified into: late night (00:00–3:00), before dawn (3:00–6:00), morning (6:00–11:00), noon (11:00–13:00), afternoon (13:00–18:00), evening (18:00–21:00), and night (21:00–00:00). The percentages of the tweets collected during these time periods were 3.5%, 3.5%, 20.7%, 10.6%, 28.0%, 20.7%, and 13.0%, respectively. The days of the week were divided into weekdays (Mon., Tue., Wed., Thurs. and Fri.), and weekend (Sat. and Sun.). 

### 2.2. Data Analysis

We used the IBM Watson Alchemy application program interface (API) [45] to conduct sentiment analysis of the collected tweets, which has been trained on billions of web pages and can provide cloud-based natural language processing. The Alchemy API introduces a combined use of linguistic analysis, which considers a sentence’s composition, and statistical analysis, which handles noisy content. Its sentiment analysis is built upon machine-learned patterns to predict the intended sentiment from text. The Alchemy API has been verified as one of the best sentiment classification tools, especially for tweets [46]. Alchemy API’s sentiment analysis could reach an accuracy of 88.36% and 86% for 2100 hotel reviews from TripAdvisor [47], and a corpus of 5370 tweets on tourism [48], respectively. The calculated sentiment score has a continuous value range of [−1, 1], referring to the polarity from extremely negative to extremely positive. Zero means the sentiment is neutral. 

Bar charts were used to graphically depict the distributions of average sentiment scores per different influencing factors. We also used the net sentiment rate (*NSR*) to judge the overall attitudes expressed by users through comparing the rate of positive and negative tweets (Equation (1)). The prevalence of users’ sentiment could be clearly revealed by looking at both the score distributions and the *NSR*:

(1) $$NSR=\;\frac{Positive\;Tweets-Negative\;Tweets}{Total\;number\;of\;Tweets}$$

Heat maps were used to present the mobility pattern of users, by using the open-source statistical package R version 3.4.0 (R Project for Statistical Computing, Vienna, Austria). The “plyr” package was used to count the number of users across land use categories and time periods. The heat maps were plotted out by using the “ggplot2” package, and further polished by using the “ggthemes”, “scales” and “viridis” packages.

In addition to the descriptive analysis, a multivariate linear mixed-effects model was used to quantify the effects of land use and time period on the sentiment score of the individual user. Analyses were performed by using the “nlme” package in R. The regression model is:

(2) $$y_{i,j,k,l}=\beta_{0}+\beta_{1}*L_{1}+\cdots +\beta_{7}*L_{7}+\beta_{8}*D_{1}+\cdots +\beta_{13}*D_{6}+\beta_{14}*T_{1}+\cdots +\beta_{19}*T_{6}+b_{0,i}+\varepsilon_{i,j,k,l}$$

where *y_i,j,k,l_* is the sentiment score for user *i* in land use category *j* during time period *l* on day *k*. We fitted the model with a random effect *b*_0*,i*_ of the intercept for user *i* to account for the clustering effect of each individual user, and we included a categorical variable of land use category (*L_j_*), a categorical variable of the days of the week (*D_k_*) and a categorical variable of the time periods of the day (*T_l_*) as the fixed effects in the model. *β*_0_ is the fixed intercept; *β*_1_–*β*_7_ are the fixed effects of other land use categories compared to farmland; *β*_8_–*β*_13_ are the fixed effects of other days of the week compared to Wednesday; *β*_14_–*β*_19_ are the fixed effects of other time periods of the day compared to before dawn; and $\varepsilon_{i,j,k,l}$ is the vector of random errors.

## 3. Results

**Figure 2:**
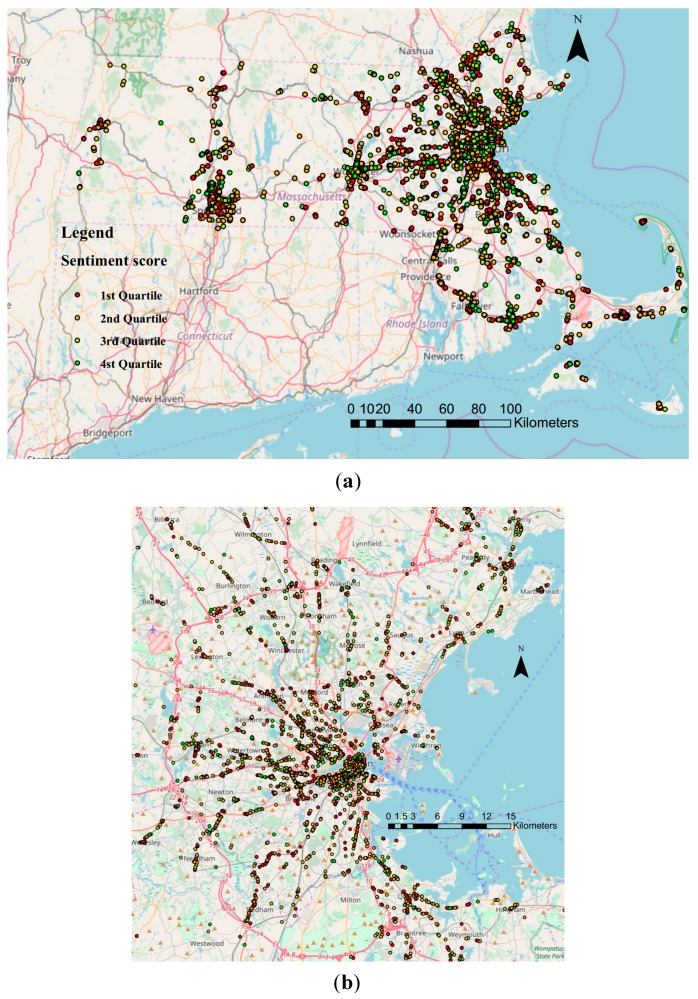
Geographical distribution of tweets with quartiles of sentiment scores in (**a**) Massachusetts and (**b**) Greater Boston area. First quartile (−1.000 to −0.410), second quartile (−0.410 to 0.000), third quartile (0.000 to 0.514), and fourth quartile (0.514 to 1.000).

Figure 2 displays the geographical distributions of the collected tweets with quartiles of sentiment scores. Most of the tweets were posted within urban/sub-urban centers, such as the Greater Boston Area, Springfield, Worcester, and Pittsfield. Seventy-eight percent of the collected tweets expressed either positive (36%) or negative (42%) attitudes from users, while the other 22% of tweets conveyed neutral sentiment. On average, the users’ sentiment conveyed by these tweets tended to be skewed to slightly negative with an average score of −0.053 and an *NSR* of −0.065.

**Figure 3:**
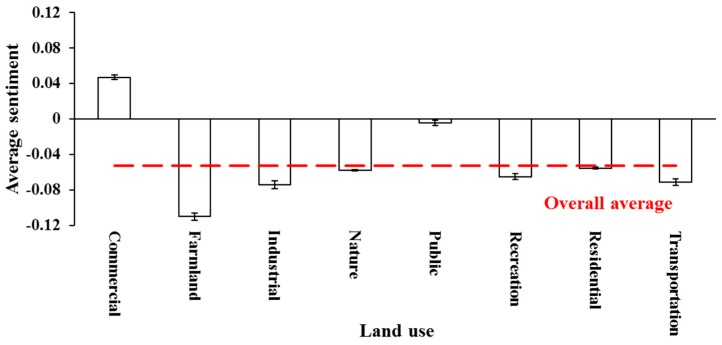
Average sentiment scores by land use categories.

**Figure 4:**
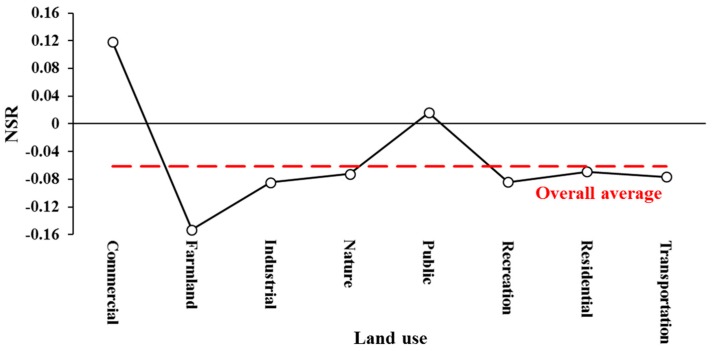
*NSR*s by land use categories. *NSR*: net sentiment rate.

Figure 3 shows the distribution of average sentiment scores across the eight land use categories. Commercial land use was the only area where sentiment scores were skewed to be positive, with 37% more positive tweets than negative tweets. The lowest average sentiment score was found in the area of farmland, with nearly 50% more negative tweets compared to positive tweets. Another interesting finding was that the positive and negative scores were almost evenly distributed in the public area. As shown in Figure 4, the variation trend of normalized polarity was highly relevant to the trend of average sentiment scores by land use categories. The commercial area was mainly concentrated in urban regions, where users showed more positive emotions with an average sentiment score of 0.047 and a *NSR* of 0.099. The overall polarity tended to be neutral in the public area with a *NSR* of 0.010. The average sentiment scores were approximately around the overall average score (−0.053) in the areas of residence, nature, and recreation. Users were more likely to show negative emotions within the areas of transportation, industry, and farmland. The spatial variations of the average sentiment scores indicated that users’ emotions could probably be affected by the surrounding environments.

**Figure 5:**
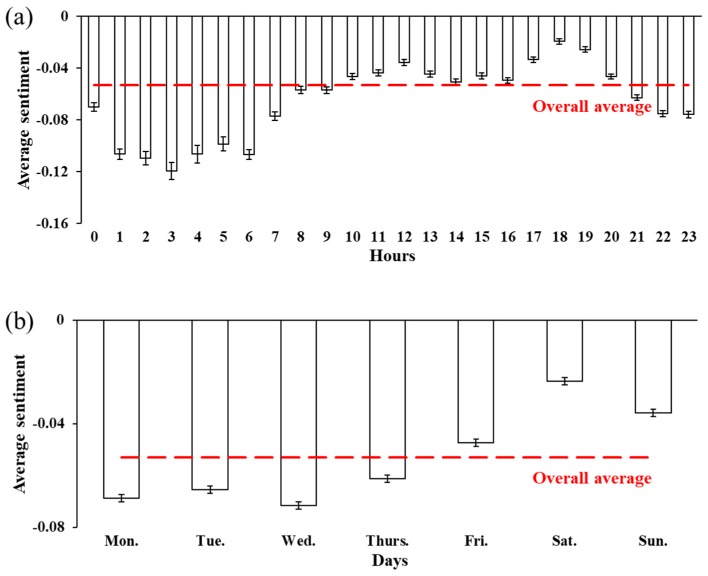
Average sentiment scores by (**a**) hours of the day and (**b**) days of the week.

**Figure 6:**
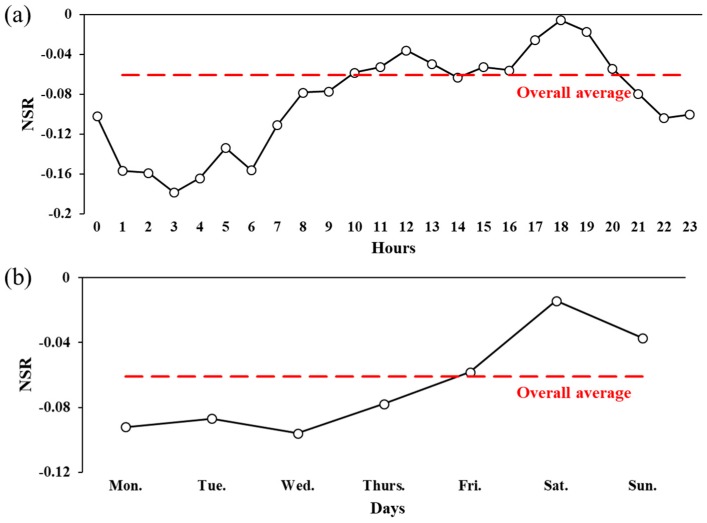
*NSR*s by (**a**) hours of the day and (**b**) days of the week.

The sentiment scores showed clear temporal patterns by hours of the day and days of the week, as shown in Figure 5 and Figure 6. It is clear that users’ sentiment followed a certain temporal pattern throughout the day. From late night to early morning, the average sentiment scores were much lower than the overall mean score, reaching the lowest at 3:00 with a value of −0.119 and an *NSR* of −0.179. The sentiment scores began to increase from noon and showed two peaks during 11:00 to 13:00 and 17:00 to 20:00. The average sentiment scores during 10:00 to 20:00 were higher than the overall mean score. The average users’ sentiment was obviously lifted on the weekend, with a mean score of −0.030 and an *NSR* of −0.026. The average sentiment score decreased to −0.063 on weekdays, along with an *NSR* of −0.082. The average sentiment scores were generally lower than the overall average among weekdays, reaching the lowest on Wednesday with a value of −0.072 and an *NSR* of −0.096, while the highest was on Friday, with a value of −0.047 and an *NSR* of −0.058. 

The temporal patterns of users’ sentiment could be partially explained by the daily routine of the general public. During the day, the two peaks of sentiment scores were during 11:00 to 13:00 and 17:00 to 20:00, which were the typical time periods for relaxation, dining, or hanging out. The most negative sentiment appeared around 3:00 when users possibly felt more emotional or anxious because of staying up around midnight. Users also tended to be unhappy when getting up early in the morning. The weekly trend shows a clear “mid-week dip” and a “weekend peak” for users’ sentiment, quite consistent with the findings by another Twitter-based linguistic inquiry study [41]. This can be interpreted as the weekend’s recovery effects on working pressure, as indicated by the lifted sentiment score on Friday.

**Figure 7:**
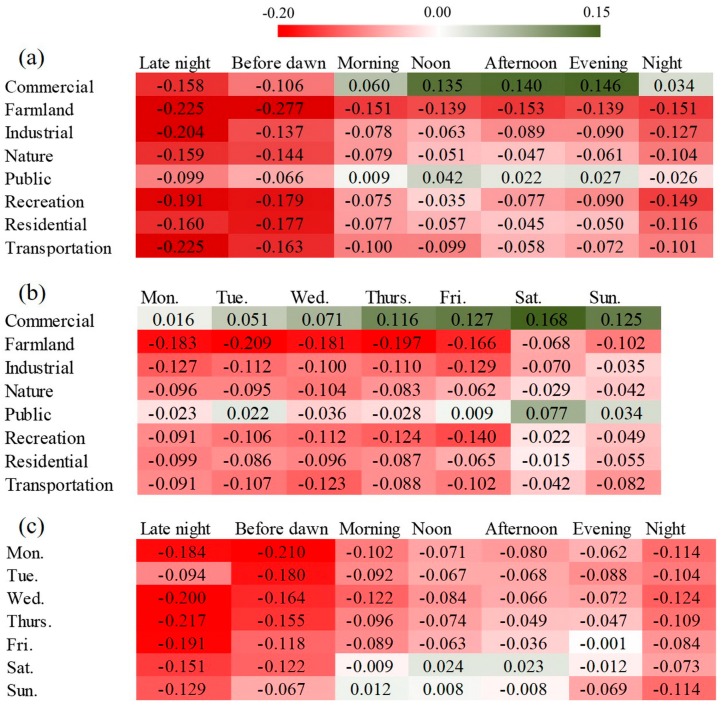
*NSR*s by (**a**) land uses and times of the day; (**b**) land uses and days of the week; and (**c**) times of the day and days of the week.

Figure 7 shows the spatiotemporal variations in the users’ sentiment. Figure 7a,b show that the *NSR*s varied greatly by different time periods in each land use category. The variation trends of *NSR*s were consistent with the temporal patterns shown in Figure 6. The overall sentiment was higher from noon to evening, and lower during midnight to early morning, the same as in all of the land use categories. The *NSR*s were also obviously higher on the weekend compared to weekdays in all the land use categories. From another perspective, the *NSR*s also varied by different land use categories during each time period. The variations of *NSR*s by land use were also consistent across all the time periods, following the trend in Figure 5. As shown in Figure 7c, the *NSR* reached a peak during the evening and went down to a bottom during the midnight every day throughout a week. The effects of land use and time period were likely to be additive of the users’ sentiment.

Except for the descriptive analysis, we used a multivariate linear mixed-effects model to quantify the influence of land use and time period on the sentiment scores, accounting for the clustering effect of users. The coefficients of each fixed variable shown in Table 2 were already modified by the random effects of users and other fixed effects. The category with the lowest average sentiment score was selected as the referent for each categorical variable. Therefore, these coefficients indicated the relative effect intensities of different variables. The interaction between variables could be neglected due to their very low cross-correlation coefficients. The model results were generally consistent with the descriptive analysis above. The results clearly show the significant intra-category differences in the average sentiment score for all the categorical variables. The average sentiment scores were obviously higher in the commercial and public areas, during the weekends, and between noon and evening, compared to their respective references. The regression model was also used to compare the users’ sentiment in certain land use category and time period. For example, the average sentiment score was increased by a value of 0.148 (*p* < 0.0001) in the commercial area during Saturday evening, compared to the score on farmland before dawn on Wednesday.

## 4. Discussion

This paper presents a case study of using Twitter-based sentiment analysis to understand public happiness and well-being. Both descriptive and statistical analyses were used to study the associations between users’ sentiment scores and different land uses and time periods. The findings show distinctive traits of the users’ sentiment across different land use categories and time periods in MA. Such an approach could, therefore, provide an economical way to gather information about the subjective QoL of a large population that is not obtainable at a grand scale from traditional survey approaches. The ability to quantify public sentiment across time and space could be of great value to government officers, urban planners, retailers, and marketers. For example, using this type of analysis can help improve the subjective QoL across different areas by better planning of land use in cities and regions. Assessment of spatiotemporal patterns of public sentiment can be considered as the initial step to formulate appropriate public policies or marketing methods in different cities and regions. On the other hand, it can also be used for evaluating the implementation of measures for improving the subjective QoL. 

**Figure 8:**
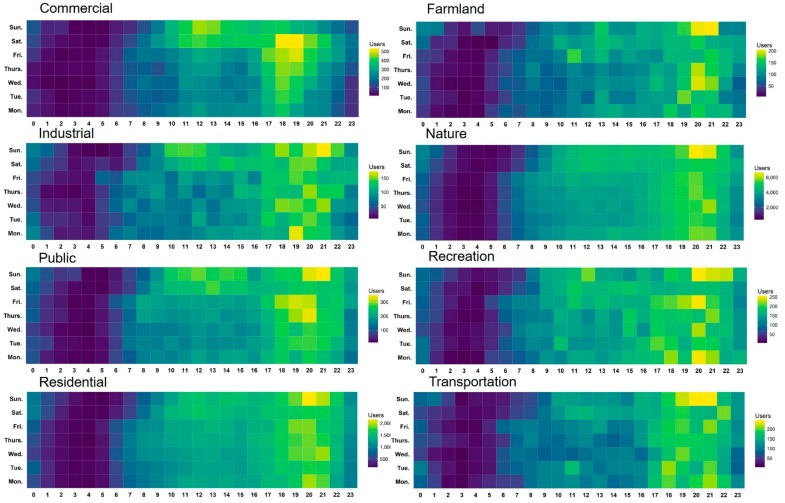
Temporal frequency of active users by land use categories.

Figure 8 shows the mobility patterns of users by land use categories. In general, the two peaks of user activity were observed during the lunchtime and the evening hours, similar to the findings of a previous study [9]. Twitter users were most active from 18:00 to 21:00 (27% of the tweets were posted), especially in the commercial area. It is also shown that users were more active during the weekend compared to the weekdays. Nevertheless, the number of active users was obviously lower in the early morning on the weekend. Different land use categories also manifested distinctive patterns. In the commercial and public areas, users were obviously more active in the daytime during the weekend than weekdays. In the areas of nature and residence, users showed regular activity patterns. The land use category of nature does not necessarily mean remote areas. In contrast, a large portion of natural land use types are located in the suburban areas near green land or water, where a large number of local residents live. This could be the reason for the similar patterns of users’ sentiment and activity in the natural and residential areas. For the other land use categories, the temporal frequency of users generally followed the overall trend, but showed more irregular patterns.

**Figure 9:**
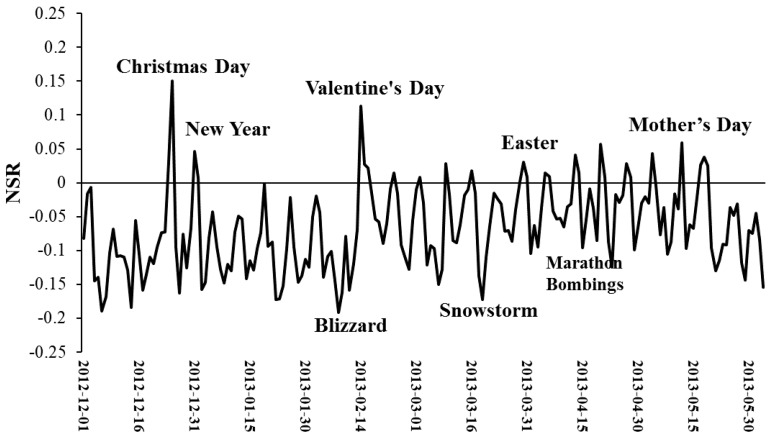
Variations of *NSR*s during the investigated date range.

The date or seasonal pattern of sentiment is not explicitly discussed in this paper, because it is more likely to be associated with social events, public holidays, and climate environments. Figure 9 shows the variations of sentiment over the investigated date range. As shown in the figure, the users’ sentiment was very sensitive to the date when it is collected. It is clear that extreme *NSR* values were highly correlated with important social events, extreme weather, and public holidays. For instance, users showed obviously more positive sentiment on Christmas, New Year, Valentine’s Day, Easter, and Mother’s Day, etc. The extremely negative sentiment was likely to be caused by bad weather or certain social events, e.g., the Boston Marathon Bombings. After the bombings, the average sentiment score soon returned to a peak value when the second suspect was arrested on 20 April 2013.

There are several limitations to this research. We used the main categories of land use due to the insufficient sample size of tweets in many sub-group types. The underlying variations of sentiment across nuanced land use types may be overlooked if just studying the eight main categories. Moreover, the intensively urban/suburban usage (Figure 2) indicates that our results are more representative of the population in non-rural regions. A larger and more widely-distributed set of tweets is necessary to study the influence of land use types on public sentiment at a finer spatial scale. The mobility of Twitter users is also a confounder of our analysis. It could be possible that the users’ sentiment were less relevant to the posting location due to their mobility. However, using a big data approach and treating the user as a random effect in linear modeling may help reduce this kind of confounding effect.

The interpretation of Twitter-based results also needs to be cautious. Opinions conveyed by users are strongly influenced by their characteristics. Twitter is a means of spreading information publicly, thus, the information and the nature of how it is expressed vary among users. Most of the variations in subjective well-being are still attributable to individual characteristics [32,33]. We considered the clustering effect of users in our modeling analysis, but that only represented a small part of users’ characteristics. The marginal R-squared value of fixed effects is 0.7% and the conditional R-squared value of the model is 10.4% for both the fixed and random effects. Therefore, the proposed model was not applicable for prediction. Instead, the model was mainly used to find out the intra-category differences in average sentiment scores. 

The demographic and social-economic characteristics of individual users are also strongly associated with the variations in what is communicated through Twitter [2]. A case study in Chicago [49] indicates that the demographic information, particular the race/ethnicity group, significantly affects the urban mobility patterns of Twitter users. As Twitter does not require users to record detailed personal information, it is not possible to obtain personal characteristics conclusively. A recent study showed that the age and gender characteristics could be inferred from 32,000 unique forenames representing over 17 million individuals in Britain [50]. This kind of approach may not cover all the Twitter users because a large portion of users prefer to use nicknames instead of full names. Demographic and neighborhood socio-economic characteristics of users can be approximately assigned according to the social census statistics [16]. However, the assigned characteristics may be biased due to the strong mobility of users. Further studies are needed to address the relationships between these individual differences and the spatiotemporal variations in users’ sentiment. In addition, the use of the Twitter service is selective. This leads to a major limitation of Twitter-based analysis that the sampled population may not be completely representative of the population of interest. Twitter users only account for 15% of Internet-using adults, mostly including young adults, African Americans, urban/suburban residents, and mobile users [51]. Moreover, only the geo-tagged tweets that users would like to share publicly can be used for this type of study, which implicates bias from the selective disclosure of information and location. Therefore, it should be noticed that the Twitter-based research results are unable to represent the total population in the studied region.

Finally, a tweet is challenging to be classified as the content is restricted to 140 characters, while usually including nuanced or ambiguous words. Some users may even convey opinions with bi-polarity. We manually verified the Alchemy API using 500 randomly-selected tweets (170 positive, 160 neutral, and 170 negative) from our Twitter dataset, and the overall accuracy can reach 80.6%. The identification accuracy for positive, neutral, and negative tweets were 82.9%, 87.3%, and 72.1%, respectively. The Alchemy API works very well in the identification of neutral polarity, but sometimes confuses the negative and positive opinions. Using a large sample size of tweets may help reduce the uncertainties in the average sentiment scores. Moreover, the complex emotional state of human may not be completely expressed using a one-dimensional sentiment scale. Human emotions can be further classified into eight types: anger, fear, joy, sadness, disgust, surprise, trust, and anticipation [52]. Further studies are recommended to analyze these emotions conveyed from tweets to gain a deeper insight of public sentiment.

## 5. Conclusions

This study clearly revealed the spatiotemporal variations of users’ sentiment within MA, based on nearly one million randomly-collected tweets during a half year. The users’ sentiment was significantly higher in the commercial and public areas, during the noon/evening and on the weekend. In contrast, users were more likely to show negative sentiment within the areas of farmland, transportation, and industry, around midnight and on weekdays. The multivariate linear mixed-effects model showed that the average sentiment score could be increased by a value of 0.148 in the commercial area during Saturday evening, compared to the score on farmland before dawn on Wednesday. The results are not conclusive due to an insufficient sample size, lack of user information and generalized classification of land use. However, the demonstrated approach can be further used to investigate public happiness and well-being in cities and regions with more comprehensive datasets.

## Figures and Tables

**Table 1:** Classification of land use in MA.

Category	Land Use Type	Number of Tweets
Commercial	Commercial	35,610
Farmland	Cranberry Bog, Cropland, Orchard, Pasture	14,951
Industrial	Industrial, Junkyard, Mining, Powerline/Utility, Waste Disposal	14,292
Nature	Brushland, Forest, Forested Wetland, Non-Forested Wetland, Open Land, Saltwater, Sandy Beach, Saltwater Wetland, Water	567,086
Public	Urban Public/Institutional	27,786
Recreation	Golf Course, Marina, Participation Recreation, Spectator Recreation, Water-based Recreation	21,365
Residential	High Density Residential, Low Density Residential, Medium Density Residential, Multi-Family Residential, Very Low Density Residential	181,577
Transportation	Transportation	17,981
Excluded	Cemetery, Nursery, Transitional	289

**Table 2:** Summary of fixed effect estimates on sentiment scores.

Variables		Coefficients of Fixed Effects	Standard Error	*p*-Value
Intercept		−0.111	0.007	<0.0001
Land use	Commercial	0.064	0.006	<0.0001
	Farmland	0.000 (referent)	-	-
	Industrial	0.021	0.008	0.0082
	Nature	0.026	0.006	<0.0001
	Public	0.037	0.007	<0.0001
	Recreation	0.022	0.007	0.0022
	Residential	0.026	0.006	<0.0001
	Transportation	0.016	0.007	0.0302
Days of the week	Mon.	0.004	0.002	0.0345
	Tue.	0.007	0.002	0.0004
	Wed.	0.000 (referent)	-	-
	Thurs.	0.006	0.002	0.0042
	Fri.	0.014	0.002	<0.0001
	Sat.	0.030	0.002	<0.0001
	Sun.	0.030	0.002	<0.0001
Times of the day	Late night	0.012	0.004	0.0044
	Before dawn	0.000 (referent)	-	-
	Morning	0.034	0.003	<0.0001
	Noon	0.044	0.003	<0.0001
	Afternoon	0.046	0.003	<0.0001
	Evening	0.054	0.003	<0.0001
	Night	0.040	0.003	<0.0001
